# Primary Langerhans cell sarcoma in the urinary bladder: Case report and literature review

**DOI:** 10.3389/fonc.2023.1118222

**Published:** 2023-03-09

**Authors:** Yongbao Wei, Huaishan Hong, Haijian Huang

**Affiliations:** ^1^ Shengli Clinical Medical College of Fujian Medical University, Fuzhou, China; ^2^ Department of Urology, Fujian Provincial Hospital, Fuzhou, Fujian, China; ^3^ Department of Pathology, Fujian Provincial Hospital, Fuzhou, China

**Keywords:** Langerhans cell sarcoma, urinary bladder, TURBT, chemotherapy, oncological outcome

## Abstract

Langerhans cell sarcoma (LCS) is a rare malignancy of dendritic cells and usually results in a poor oncological outcome. Thus, LCS is usually given a positive administration. Herein, we presented the first case of primary LCS in the urinary bladder staged T1N0M0 and treated by TURBT and short-term local chemotherapy. Our experience in this unique case may suggest that LCS in the urinary bladder with a non-muscle-invasive stage may be managed according to the treatment model of non-muscle-invasive urothelial carcinoma of the urinary bladder.

## Background

Bladder cancer is a common genitourinary malignant tumor with urothelial carcinoma as the main type (1). The treatment modality is currently based on TNM staging, and the surgical treatments of non-muscle-invasive (NMIBC), muscle-invasive (MIBC), and postoperative adjuvant therapy vary differently (1). In addition to conventional surgical treatments such as transurethral resection of bladder tumor (TURBT), regular cystoscopy and postoperative adjuvant bladder irrigation chemotherapy or immunotherapy are required for NMIB (1, 2). For MIBC and recurrent NMIBC, radical cystectomy is one of the standard treatment modalities, which can be performed by a robot-assisted, laparoscopic, or open approach (3). Overall, these three treatment approaches’ short- and long-term outcomes differ and require effective communication with patients (4).

Langerhans cell sarcoma (LCS) is a rare malignancy of the epidermis’s dendritic cells involved in multiple-organ recidivism (5). It is characterized by cytological atypia, frequent mitoses, and aggressive clinical behavior and usually results in poor oncological outcomes (5, 6). We present the first case of primary LCS in the urinary bladder, with early results that look extremely promising. TURBT and bladder irrigation chemotherapy were performed. No local tumor recurrence was observed during the 3 years of follow-up. The approval for this study was obtained from the Institutional Review Board of Fujian Provincial Hospital. Written informed consent was obtained from the guardians of the patient.

## Case presentation

**Figure 1:**
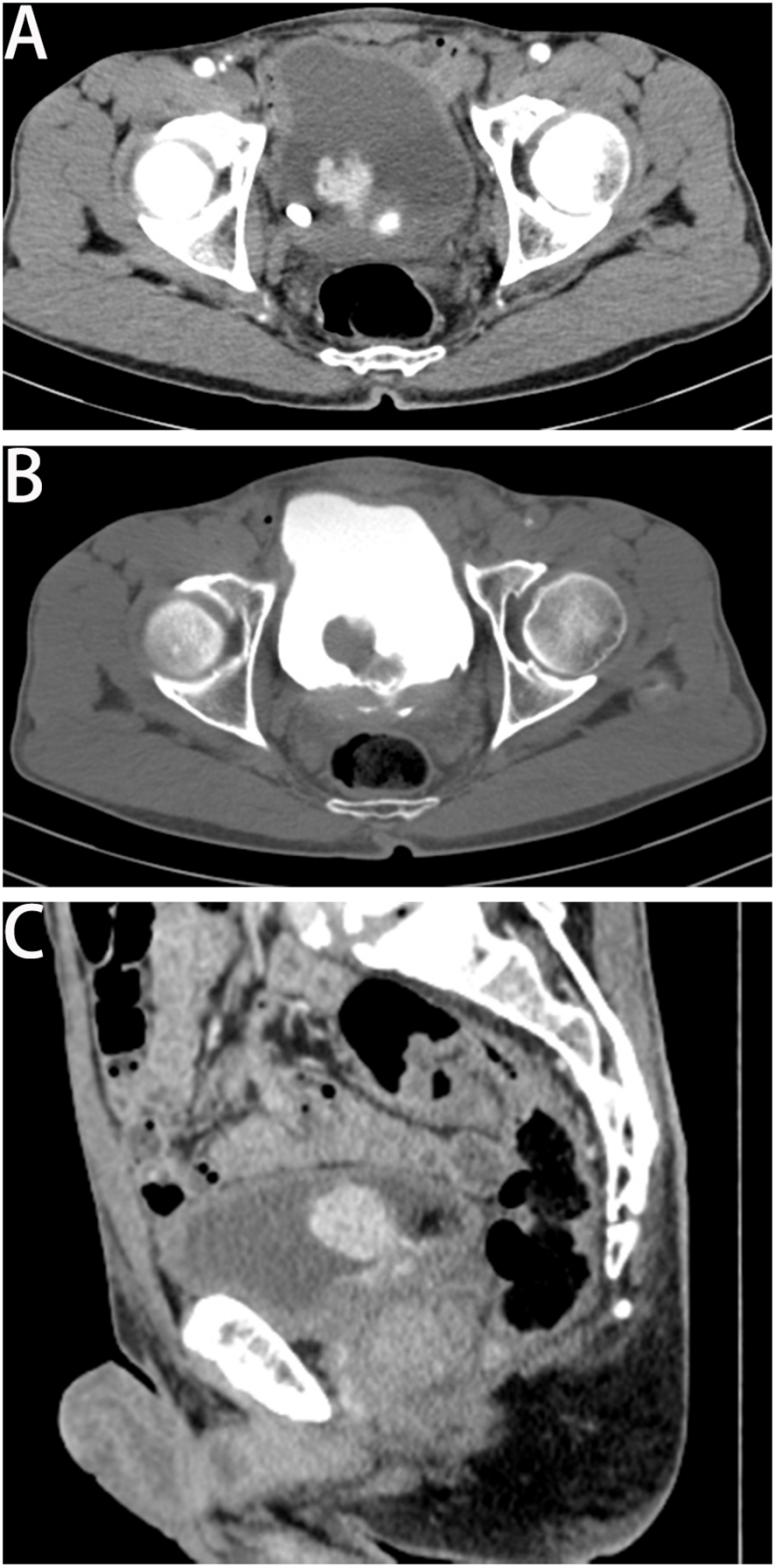
Results of computed tomography urography. Cross sections: panel **(A)** (arterial phase) and panel **(B)** (delay 2 phase). Sagittal section: panel **(C)** showed a lobulated tumor located in the bladder’s posterior wall with its clear boundary. The size was approximately 3.5 × 2.4 × 2.1 cm. The enhanced CT scan indicated that the tumor was significantly enhanced.

**Figure 2:**
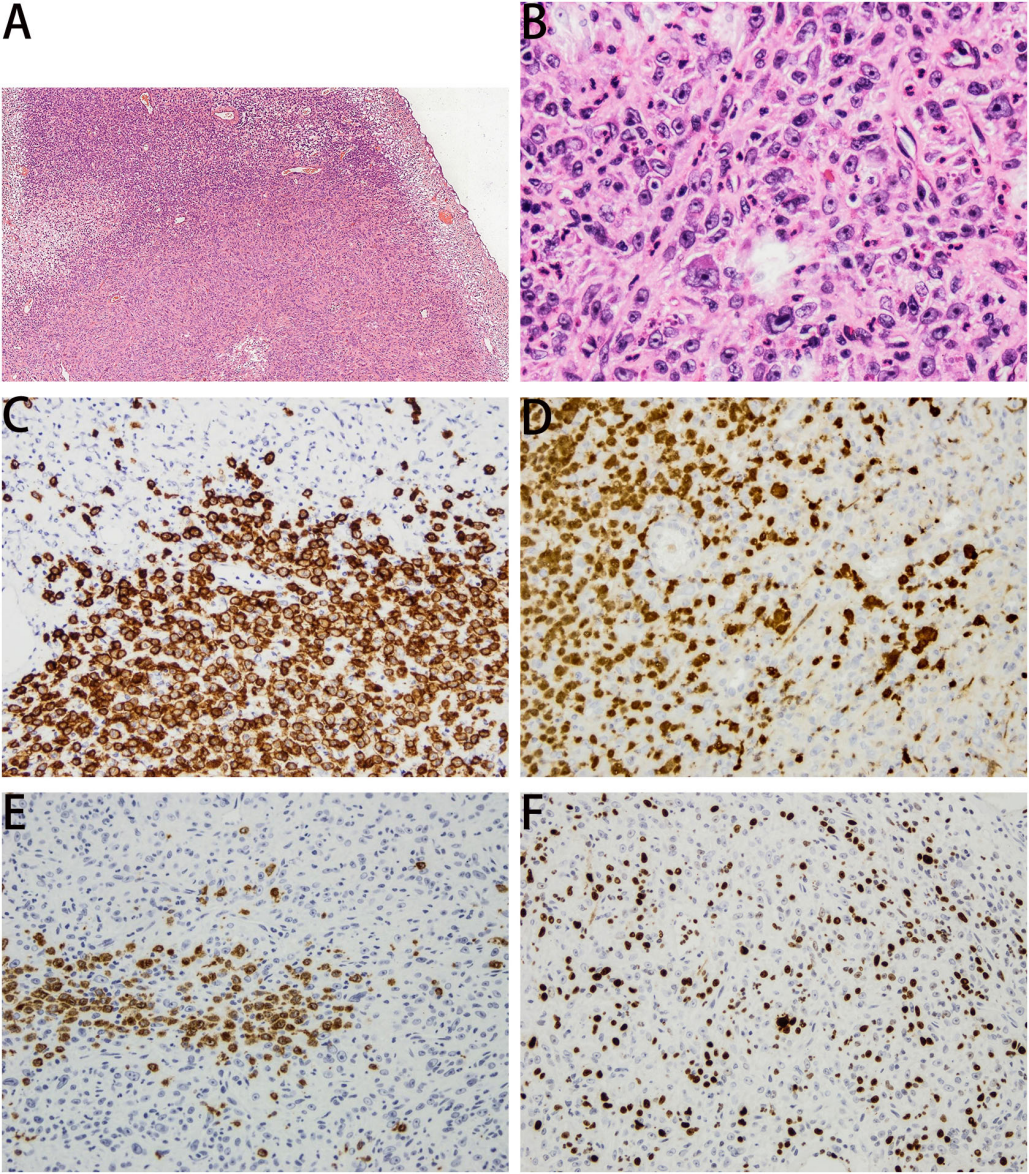
Panel **(A)** (HE, ×40) and panel **(B)** (HE, ×200) showed that the tumor cells were epithelioid, spindle-shaped, and vacuolar in the nucleus. The nucleolus was visible, and the nuclear membrane was clear. Immunohistochemical results are as follows. Panel **(C)** CD1a (×100, ++), for sarcomatoid area +. panel **(D)** S100 (×100, ++), for sarcomatoid area +. panel **(E)** CD27 (×100, ++), for sarcomatoid area +. panel **(F)** Ki67 (35%).

A 63-year-old man was admitted to our hospital for urinary urgency and frequency for 6 months, accompanied by a urinary interruption. He complained of no gross hematuria or other discomfort. His medical history is not unique. Ultrasonography examination revealed a substantial mass located in the bladder and also two small stones. After admission, computed tomography (CT) showed a lobulated tumor located in the bladder’s posterior wall with a clear boundary. The size was approximately 3.5 × 2.4 × 2.1 cm. The enhanced CT scan indicated that the tumor was significantly enhanced (**Figure 1**). The patient was likely to be diagnosed with NMIBC based on preoperative examinations. Thus, TURBT was performed to remove the tumor and obtain an exact pathological and staging diagnosis. During the operation, the tumor was located on the left posterior wall, with around 1.5-cm pedicle, like a cauliflower. The postoperative pathological results showed that the tumor cells were epithelioid, spindle-shaped, and vacuoles in the nucleus. The nucleolus was visible; the nuclear membrane was transparent; karyolysis, focal necrosis, and also rich blood vessels could be seen; and some interstitial inflammatory cells were seen infiltrated. In combination with immunohistochemistry, LCS was finally confirmed (**Figure 2**). Immunohistochemical results showed positive expressions of S100 (++, sarcomatoid area: +), CD27 (++, sarcomatoid area: +), CD1a (++, sarcomatoid area: +), Ki67 (35%), CD68 (++), and CD163 (++). Negative expressions were shown in ALK Actin (SM), Desmin, ALKp80, HMB45, Malan-a, CK (pan), Gata-3, CD34, CD30, CD20, CD3, CD21, CD99, CD56, EMA, sox10, CD56, SY, and C123. No tumor invasion of muscular bladder tissue was found. The postoperative staging was made as T1N0M0, a high-risk NMIBC. The patient was treated twice with 50 mg of epirubicin for bladder irrigation, once within 24 h after surgery and the other 1 week after surgery. After the pathological diagnosis is confirmed, since there is no empirical reference for bladder LCS treatment, we recommend Bacillus Calmette-Guerin (BCG) for adjuvant therapy according to the treatment guidelines for high-risk NMIBC (1, 7). However, patients refused BCG therapy due to drug accessibility and economic reasons. In addition, the patient did not cooperate with other treatments and rigorous follow-up planning. Fortunately, the patient had no significant discomfort after 3 years of follow-up. Both cystoscopy and evaluation of exfoliative cytology specimens revealed no tumor recurrence.

## Discussion

Langerhans cell sarcoma (LCS) is a rare malignant tumor of Langerhans cells; less than 70 cases have been reported (5). The age of onset of LCS is more extensive, including adults and children. LCS is characterized by multiple-organ recidivism, including skin, lymph nodes, liver, spleen, lungs, and bone (5). According to the previous systematic review (5), our case of LCS is the first case reported primarily in the urinary bladder.

The distinguished diagnosis should be made between LCS and Langerhans cell histiocytosis (LCH) (5). LCH is a clinically benign disease and rarely transforms into LCS (6). The Langerhans cells are distinguished by their morphology and are a positive expression of CD1a, S100, CD21, CD35, and CD68 (8). A recent study found that B7-H1, B7-H3, and B7-H4 were expressed on Langerin+ tumor cells and may also be potential biomarkers to identify LCS (9). The diagnosis of LCS is only confirmed by pathological examination, based on the malignant cytological features, number of mitoses present, and immunohistochemical profiles (5). Our case fit those characterized descriptions of Langerhans cells and was also positive in the distinctive immunohistochemical profiles.

The clinical spectrum of changes in LCS is vast and can develop into highly invasive lesions, often leading to patient death. However, given LCS rarity, there is a lack of evidence regarding the most appropriate treatment for this disease. 52% of patients with LCS were managed by a single modality, compared with 42% treated by combination therapy (5). The role of surgery is essential and was used in 47.0% (31/66) LCS cases and was the sole intervention in 14% (9/66) of them (5). Other treatment modalities include adjuvant therapy, chemotherapy, radiotherapy, and bone marrow transplant (5). Up to date, the overall 5-year disease-specific survival (DSS) for all LCS patients was 28%, whereas in the case of single-organ involvement, the 5-year DSS was 70% (5). This means the oncological outcome would be much better if LCS is only a single focal lesion. The present case is only a single focal lesion and primarily occurred in the urinary bladder. After TURBT treatment for this patient, only twice epirubicin for bladder irrigation therapy was performed without any other treatment. No local tumor recurrence was observed during the 3 years of follow-up. Furthermore, Kawase et al. (10) found that CD56-positive LCS showed invasive clinical behavior and poor prognosis. In our case, the patient was negative for CD56, suggesting a good prognosis. In the present case, we preferred intravesical BCG instillation as postoperative adjuvant therapy, which is the first choice of postoperative treatment for high-grade NMIBC to reduce recurrence rates and risk of progression (7). However, for NMIBC, several intravesical treatments can reduce the risk of recurrence compared with TURBT alone, in which BCG is considered the only drug associated with a reduced risk of progression but may have a higher risk of adverse events compared with other intravesical treatments (11). However, when BCG or mitomycin C shortage happens, several other intravesical chemotherapies may be considered, including gemcitabine and epirubicin (12). Due to drug accessibility and economic considerations, the presented case only undertook twice adjuvant epirubicin irrigation. Fortunately, no signs of tumor recurrence happened until 3 years after surgery, even without regular follow-ups.

As a case report, our study had significant limitations to the value of evidence-based medicine. First, given the rarity of LCS originating in the bladder, we could only provide a unique case to share our experience. Therefore, our experience might give a little reference value, and we needed to be vigilant that LCS in other body parts is fatal cancer, although our case had no tumor recurrence 3 years after treatment, which did not mean that the lethal characteristics of LCS primarily in the bladder would reduce. Thus, we are still vigilant and should consider favorable comprehensive treatment and strict follow-up for such patients. In addition, we presented a T1N0M0 stage of LCS and shared our treatment experience; we could not anticipate the oncological outcomes of a more aggressive cancer stage (MIBC or an advanced one) and other appropriate treatment modalities, including surgery and management of medications.

## Conclusions

The present case is the first report of primary LCS in the urinary bladder. The diagnosis of LCS in the urinary bladder should be made according to pathological examination. Our experience in this unique case may suggest that LCS in the urinary bladder with a non-muscle-invasive stage may be managed by transurethral resection of the bladder tumor and local chemotherapy, as well as close follow-up.

## Data availability statement

The original contributions presented in the study are included in the article/supplementary material. Further inquiries can be directed to the corresponding authors.

## Ethics statement

The studies involving human participants were reviewed and approved by Fujian Provincial Hospital. The patients/participants provided their written informed consent to participate in this study. Written informed consent was obtained from the individual(s) for the publication of any potentially identifiable images or data included in this article.

## Author contributions

YW wrote the paper, HSH dealt with the case, HJH did the pathological analysis. All authors contributed to the article and approved the submitted version.
